# Resting-state EEG microstates link neural dynamics to fluid intelligence in mild cognitive impairment

**DOI:** 10.3389/fnagi.2026.1734828

**Published:** 2026-05-20

**Authors:** Ceyhun Sayman, Eren Toplutaş, Uğur Aylak, Shair Shah Safa, Mehmet Savcılı, Ayşe Karakuş, Doğukan Uçak, Lütfü Hanoğlu, Seyda Cankaya, Adil Mardinoglu, Burak Yuluğ

**Affiliations:** 1 Department of Neurology, Alanya Alaaddin Keykubat University, Antalya, Türkiye; 2 Institute of Health Sciences, Department of Neurology, Translational Neurodevelopmental Medicine PhD Program, Istanbul University, Istanbul, Türkiye; 3 Department of Neurology, Istanbul Medipol University, Fatih, Türkiye; 4 Brain and Cognition Research Center (BEYKOG), Istanbul Medipol University, Istanbul, Türkiye; 5 Graduate School, Department of Neuroscience, Bahcesehir University, Beşiktaş, Türkiye; 6 Neuroscience Research Center, Research Institute for Health Sciences and Technologies (SABITA), Istanbul Medipol University, Istanbul, Türkiye; 7 Centre for Host-Microbiome Interactions, Faculty of Dentistry, Oral & Craniofacial Sciences, King’s College London, London, United Kingdom; 8 Science for Life Laboratory, KTH Royal Institute of Technology, Stockholm, Sweden

**Keywords:** Benton Facial Recognition Test, EEG microstates, fluid intelligence, mild cognitive impairment, Raven’s Matrices, resting-state EEG

## Abstract

**Background:**

Resting-state EEG microstates offer millisecond-scale markers of large-scale network dynamics that may capture early cognitive dysfunction in Mild Cognitive Impairment (MCI). We investigated whether microstate characteristics relate to global cognition and fluid intelligence in MCI.

**Methods:**

Sixty adults (30 MCI; 30 healthy controls, HC) were evaluated using the Mini-Mental State Examination (MMSE), Montreal Cognitive Assessment (MoCA), and Raven’s Progressive Matrices (RPM), and the Benton Facial Recognition Test (BFRT). Resting-state, eyes-closed EEG was recorded, and microstate Global Explained Variance (GEV) was calculated by mapping study specific topographies to the seven canonical classes (A–G) of the Metamaps2023 templates. In addition to GEV, temporal parameters (duration, occurrence, and coverage) were analyzed. Group differences and age/education/sex-adjusted correlations were assessed using a unified non-parametric framework with FDR correction.

**Results:**

MCI patients showed significantly lower scores across all cognitive domains compared to HC (*p* < 0.01). GEV was significantly higher in the MCI group for microstate B (≈11.3% vs. 7.9%; *p* = 0.007) and microstate C (≈6.1% vs. 4.1%; *p* = 0.007), while other classes showed no significant differences. Temporal analysis revealed that microstates B and C exhibited significantly increased occurrence and coverage in the MCI group, whereas mean duration did not differ between groups. Correlation analysis demonstrated that increased GEV of microstates B and C was associated with poorer performance on the MoCA and RPM, while microstates A and G showed positive correlations with MMSE scores. These associations remained robust after controlling demographic covariates.

**Conclusion:**

MCI is characterized by an increased contribution of microstates B and C, which are linked to impairments in fluid reasoning and executive control. These findings suggest that microstate dynamics serve as sensitive markers of early network disorganization in the predementia stage, reflecting a loss of neural flexibility that mirrors cognitive decline.

## Introduction

### Mild cognitive impairment in aging

Mild Cognitive Impairment (MCI) is a term that encompasses clinically categorized cognitive decline that exceeds normal aging yet remains too mild to meet the criteria for dementia. Generally, MCI represents a transitional boundary between a completely healthy aging process and dementia, where the individual has experienced a notable cognitive decrease that does not lead to significant impairment in performing basic activities of daily living (Gauthier et al., 2006; Petersen, 2016). Importantly, MCI is often conceptualized as a prodromal stage of Alzheimer’s disease (AD). Longitudinal data suggest that individuals with MCI progress to dementia at a rate of approximately 10 to 15% per year, whereas cognitively healthy seniors progress at a rate of only 1 to 2% per year (Ward et al., 2013). However, since not all individuals with MCI may progress to dementia, it can be viewed as a condition of heightened risk due to the higher probability of progressing to Alzheimer’s disease (AD) or various other neurodegenerative conditions (Petersen et al., 2009; Roberts and Knopman, 2013). Consequently, MCI serves as a crucial opportunity to investigate brain alterations associated with the initial signs of memory decline, acting as a testing ground for biomarkers that could aid in predicting or postponing the emergence of dementia.

Fluid intelligence encompasses abstract reasoning, creative problem-solving techniques, and the ability to think logically without relying on prior knowledge. This cognitive skill is a key element of general intelligence and normally reaches its highest level in early adulthood, subsequently declining with age (Kent, 2017; Yuan et al., 2006). Importantly, cognitive decline in MCI extends beyond just memory issues and affects fluid intelligence as well (Manard et al., 2014). In that context, several studies have shown that individuals with MCI experience difficulties with abstract reasoning and problem-solving, even when their memory is still relatively intact. For instance, Carbone et al. (2021) discovered that individuals with early onset MCI exhibited notably poorer performance than healthy adults on Raven’s Matrices (RPM), a commonly utilized assessment for evaluating fluid intelligence (Carbone et al., 2021). RPM is a non-verbal, culture fair test that evaluates abstract reasoning and pattern recognition. It is considered one of the most reliable tools for assessing fluid intelligence because it is less influenced by language skills or formal education. Therefore, it provides a useful way to compare reasoning abilities between MCI patients and healthy individuals (Alves et al., 2018).

In addition to RPM, which assesses general non-verbal reasoning, the Benton Facial Recognition Test (BFRT) was employed to evaluate higher-order visuoperceptual processing, specifically the integrity of the ventral visual stream (Benton, 1994; Murray et al., 2022; Canário et al., 2023). While these tests assess distinct cognitive domains, their combined use provides a complementary rather than comprehensive perspective on non-verbal cognitive functioning in MCI. Specifically, RPM primarily captures abstract reasoning and fluid intelligence, whereas BFRT targets higher-order visuoperceptual processing. The primary aim of including both measures is not to fully characterize all non-verbal cognitive domains, but rather to examine whether EEG microstate dynamics are associated with both domain-specific processes and their shared dependence on large-scale neural network coordination.

Rather than conceptualizing BFRT as a component of fluid intelligence, we propose that the coordination of large-scale neural networks, as quantified through EEG microstates, underlies both the complex perceptual discrimination required for BFRT and the abstract reasoning measured by RPM (Zappasodi et al., 2019; Barbey, 2018). Consequently, alterations in microstate dynamics in MCI may signify a breakdown in this large-scale network integration. In this context, resting-state electroencephalography (EEG) provides a significant advantage as a non-invasive method for studying brain function across both healthy and diseased states. In neurodegenerative disorders such as AD, resting-state EEG consistently demonstrates a shift toward slower brain rhythms, characterized by increased delta and theta band power and decreased alpha and beta power (Babiloni et al., 2010). In addition to these spectral changes, MCI is characterized by altered anterior to posterior distribution of alpha activity and reduced functional connectivity, reflecting early synaptic dysfunction (Jelic et al., 1996; Jelic et al., 2000). These patterns are considered characteristic of the neurodegenerative process, and contemporary topographic analyses such as microstates provide more refined insight into how these rhythmic changes manifest as stable neural network dynamics.

One advanced method of EEG analysis is microstate analysis, which identifies brief periods (typically 80–120 ms) of stable scalp potential topographies known as microstates. Initially presented by Lehmann and colleagues in the 1980s, microstates are thought to represent brief activations of extensive brain networks and are frequently referred to as the “atoms of thought” because of their distinct and repetitive patterns (Lehmann et al., 1987). Microstates are sub-second patterns of global neuronal activity and are considered the “building blocks” of spontaneous cognition, providing a unique temporal resolution that other modalities, such as functional magnetic resonance (fMRI), cannot capture (Michel and Koenig, 2018). Recent studies indicate that it is possible to reliably identify up to seven unique microstate categories (Custo et al., 2017; Bréchet et al., 2019). These microstate patterns have been associated with functional brain networks and regions via EEG source localization and multimodal approaches that combine EEG with fMRI (Custo et al., 2017; Asha et al., 2024). As highlighted in a recent systematic review by Tarailis et al. (2024), specific microstate classes (A-G) are consistently linked to distinct cognitive domains, such as phonological processing, visual attention, and salience detection. The existence of open-access microstate templates described in the literature further enhances the ability to compare analyses across different investigations (Kleinert et al., 2024; Lassi et al., 2023). Also, alterations in microstate parameters including duration, occurrence, coverage, and GEV changes are present in both psychiatric as well as neurodegenerative disorders, emphasizing the clinical importance of using this time-sensitive tool to understand the organization of large-scale functional brain networks (Hanoglu et al., 2022). Previous research comparing microstate features across the dementia spectrum has shown that as patients progress from healthy aging to MCI and AD, there is often an increased contribution (GEV and/or coverage) of microstate class C and a reduced in Class D, reflecting a shift in the balance between the salience and executive control networks (Musaeus et al., 2019; Smailovic and Jelic, 2019; Tait et al., 2020). However, most studies have focused on diagnostic classification rather than the underlying cognitive architecture, leaving the link between these temporal dynamics and fluid intelligence unexplored.

The primary benefits of resting-state EEG over fMRI and positron emission tomography (PET) include its non-invasiveness, cost-effectiveness, and suitability for repeated assessments over time, thereby facilitating a longitudinal perspective on brain dynamics and their clinical relevance. Herein, EEG microstate analysis could serve as a valuable complement to conventional neuropsychological testing, particularly in detecting subtle neural inefficiencies that routine evaluations frequently overlook in predementia phases. Although EEG offers methodological benefits, including microstate analysis, for identifying electrophysiological indicators of overall cognitive decline, there is limited understanding of how EEG characteristics are connected to complex cognitive functions such as fluid intelligence in individuals with MCI (Tanaka et al., 2024). For instance, fluid intelligence involves advanced reasoning and problem solving, which may be influenced by alterations in neural networks. This relationship is suggested by the correlation observed between brain oscillatory patterns and intelligence in individuals with no cognitive impairments (Zakharov et al., 2020). To the best of our knowledge, few studies have explicitly investigated the link between resting EEG features and fluid intelligence in MCI. Since MCI can involve not only memory impairments but also executive impairments and reasoning deficits, it is important to determine whether EEG markers reflect these higher-order changes in cognition. In a related context, the connection between resting-state EEG microstate characteristics and performance on tasks measuring fluid intelligence has not been extensively investigated, especially among individuals with MCI (Lassi et al., 2023; Wijaya et al., 2023). Addressing these identified gaps in existing research, the current study aims to examine the relationship between EEG microstate dynamics and fluid intelligence in those with MCI.

In addition to group comparisons, this study employs correlation analyses within the MCI group to examine the direct relationship between temporal EEG microstate parameters (e.g., duration, occurrence, and coverage) and specific cognitive scores obtained from RPM and BFRT. While previous studies have primarily focused on using microstates for diagnostic classification (MCI vs. AD), our study extends this work by exploring the underlying neural dynamics. This approach aims to determine whether individual variations in large-scale brain dynamics can serve as a proxy for the degree of cognitive impairment within the MCI spectrum.

## Materials and methods

### Participants

The study initially included a total of 60 participants: 30 MCI and 30 healthy control (HC) included in the analysis. A total of 60 participants were included, consisting of 30 individuals with MCI (mean age = 65.47 ± 8.54 years) and 30 healthy controls (mean age = 64.50 ± 7.42 years). The groups were matched for age (*p* = 0.642) and sex (15 males/15 females per group, *p* = 1.000). While the MCI group had slightly fewer years of education, this difference did not reach statistical significance (*p* = 0.051). Detailed neuropsychological and demographic data were summarized in Table 1. All participants were evaluated by neurology consultants in the outpatient clinic of Alanya Alaaddin Keykubat University Education and Research Hospital. The clinical diagnosis of MCI was established based on Petersen’s criteria, supported by neuropsychological assessment including Mini-Mental State Examination (MMSE) and Montreal Cognitive Assessment (MoCA). Inclusion criteria for MCI participants were based on the DSM-5 criteria for Mild Neurocognitive Disorder. To ensure the absence of dementia, we strictly followed the clinical requirement that cognitive deficits must not interfere with capacity for independence in everyday activities. Objectively, MCI was defined by a MoCA score between 18 and 25, whereas HC participants were required to have a MoCA score ≥ 26. This stratification ensures a clear distinction between healthy aging, the prodromal MCI stage, and overt dementia.

**Table 1:** Demographic and neuropsychological characteristics of the MCI and HC groups.

Variable	HC (*n* = 30)	MCI (*n* = 30)	*p* value	Effect size
Age (mean ± SD)	64.50 ± 7.42	65.47 ± 8.54	0.642	−0.121
Sex (m/f)	15/15	15/15	1.000	
Years of Education (mean ± SD)	9.43 ± 4.60	7.03 ± 3.61	0.051	−0.2767
MMSE (mean ± SD)	28.90 ± 1.24	25.07 ± 2.60	**<0.001**	**−0.8489**
MOCA (mean ± SD)	23.73 ± 2.46	17.30 ± 2.61	**<0.001**	**2.533**
Benton Form F (mean ± SD)	9.63 ± 2.30	7.20 ± 2.83	**<0.001**	**0.944**
Raven’s Matris (mean ± SD)	26.37 ± 10.77	15.10 ± 6.48	**<0.001**	**1.268**

Data are presented as mean ± standard deviation. *p*-values for categorical variables (sex) were determined via chi-square tests. For continuous variables, independent samples *t*-tests (with Cohen’s d) or Mann–Whitney U tests (with rank-biserial correlation) were applied based on normality distributions. Bold values denote statistical significance (*p* < 0.05).

HC had no history of neurological or psychiatric disorders. All participants had normal vision. Exclusion criteria included the presence of epilepsy, major psychiatric illness, current use of antiepileptic drugs, or any structural brain abnormalities on imaging. All participants gave written informed consent. The study protocol was approved by the Ethics Committee of Alanya Alaaddin Keykubat University Clinical Research (Ethical Number: 05-01, Date: 12.03.2025), and written informed consent forms were obtained from participants after thoroughly informing them about the study protocol.

### Cognitive assessment

All participants were subjected to full cognitive evaluations, including examinations of general cognitive screening and fluid intelligence. The global cognitive status evaluations were carried out by MMSE and MoCA. The MMSE provided a brief assessment of orientation, memory, and language functions. In contrast, the MoCA was administered to act as a more sensitive instrument capable of detecting early impairment in the executive, attentional, and visuospatial domains (Nasreddine et al., 2005). Complementary neuropsychological tests were used to assess fluid intelligence. RPM were presented as a reputed, non-verbal test for abstract reasoning and pattern recognition and universally accepted criteria for measuring fluid intelligence (Murphy et al., 2023). The Benton Facial Recognition Test (BFRT) was included to assess higher order visuoperceptual processing, particularly functions associated with the ventral visual stream, rather than non-verbal reasoning (Benton, 1994; Putcha et al., 2019). Therefore, these techniques offered a robust, multidimensional means of assessing the cognitive functioning of MCI participants and HC and permitted further exploration of correlations with EEG microstate parameters.

### EEG recording

Functional Imaging and Cognitive-Affective Neuroscience Lab (fINCAN), Health Sciences and Technology Research Institute (SABITA), Istanbul Medipol University, 34,815, Istanbul, Türkiye Resting-state EEG data were recorded using a Nihon Kohden Neurofax EEG-1200 system (Nihon Kohden Corporation, Tokyo, Japan). EEG recordings were obtained using 19-channel Ag/AgCl electrode cap positioned according to the international 10–20 system (F3, F4, C3, C4, P3, P4, O1, O2, F7, F8, T1, T2, T3, T4, T5, T6, Fz, Cz, Pz) with A1 and A2 as linked-ear references. Participants sat comfortably in a sound-attenuated and dimly lit room for 5 min with eyes closed at the electrophysiology laboratory of Alanya Alaaddin Keykubat University Training and Research Hospital.

The signals were then amplified and digitized, with a sampling frequency of 200 Hz. During recording, the low-pass filter was set to 100 Hz and the high-pass filter to 1 Hz. Throughout the session, electrode impedance was maintained at below 10 kΩ. The choice of a 1 Hz high-pass filter during acquisition was specifically intended to minimize slow-drift artifacts and perspiration-related noise, which can significantly compromise the topographic stability required for subsequent microstate analysis (Khanna et al., 2015). Following the recommendations of Michel and Koenig (2018), a 1 Hz cut-off was chosen to ensure that the quasi-stable scalp potential topographies are not distorted by non-neural low frequency oscillations, which is standard practice in microstate research.

### EEG preprocessing

EEG preprocessing was conducted using the EEGLAB 2025.0 toolbox within MATLAB 2024b (Delorme and Makeig, 2004). Raw EEG recordings were imported in EDF format, and standard electrode coordinate information was assigned to all channels. To maintain consistency across participants and focus on the standard clinical montages, a subset of 19 scalp channels (Fp1, Fp2, F3, F4, C3, C4, P3, P4, O1, O2, F7, F8, T3, T4, T5, T6, Fz, Cz, and Pz) was extracted from the original 64-channel recordings for further analysis. Ear electrodes were removed from the analysis to eliminate confounding signals, so that the final setup consisted of 19 scalp channels for the analysis. Power line noise was removed using a 50 Hz notch filter to lessen the effect of electrical interference. A bandpass filter between 1 and 40 Hz was applied in order to keep the frequency information most associated with cognitive as well as resting-state processes suitable for microstate analysis. The signals were subsequently re-referenced to the common average reference, reducing bias from single reference electrodes (Bigdely-Shamlo et al., 2015).

The noise correction process was carried out through a defined series of automated preprocessing steps. Initially, channels that exhibited excessive noise or unstable impedance characteristics were detected and substituted using spherical spline interpolation to maintain spatial integrity; specifically, a maximum of two channels were interpolated per participant. Following this, the Artifact Subspace Reconstruction (ASR) algorithm was utilized to identify short-lived high-amplitude variations and reconstruct the impacted segments, effectively reducing non-stationary artifacts like abrupt movements or environmental interferences. The Independent Component Analysis (ICA) was conducted using the runica algorithm, which is a reliable technique for breaking down multichannel EEG data into temporally independent and spatially unique components. Components associated with eye movements (such as blinks and saccades) and muscle artifacts were recognized based on their topographic, spectral, and temporal attributes and were systematically eliminated from the dataset (Mullen et al., 2015); on average, a maximum of two independent components per subject were removed to preserve neural signal integrity.

Following automated processes, a researcher conducted a visual examination of the datasets. This final judgment allowed for the recognition and removal of leftover artifacts that the automated processes failed to correct sufficiently, thereby ensuring that the signals reflected authentic physiological neural activity. Methodology was intentionally designed to balance a high level of automation with expert monitoring to maximize reproducibility with a concomitant minimizing of spurious activity inclusion. To conduct microstate analysis, preprocessed EEG data free from artifacts for each subject was segmented into continuous segments. From the original 300 s recordings, final artifact-free segments ranged from 120 to 300 s per participant, ensuring a robust and sufficient data length for the stable estimation of microstate parameters (Nagabhushan Kalburgi et al., 2024).

### EEG microstate analysis

EEG microstate analysis was performed using the MICROSTATELAB 2.1 plugin for the EEGLAB 2025.0 toolbox running in MATLAB 2024b (Nagabhushan Kalburgi et al., 2024). For each participant, microstate maps were identified by applying the k-means clustering algorithm to Global Field Power (GFP) peaks. Prior to clustering, polarity of the EEG signal was ignored and data were normalized. This procedure ensured that only the most topographically stable time points contributed to the clustering solution (Michel and Koenig, 2018).

Following individual clustering, group-average maps were derived across all participants. These maps were then sorted and matched to the publicly available Metamaps2023 template to facilitate standardization and comparability. Outlier detection was conducted to evaluate the dependability of the clustering results, and no maps were recognized as outliers. Finally, a seven-map template solution from Metamaps2023 was applied, and participants’ individual maps were backfitted to this standardized template (Koenig et al., 2024).

For each participant, Global Explained Variance (GEV) values were computed by backfitting the standardized microstate maps to the EEG data (Murphy et al., 2024). State-wise GEV was calculated as


$$\mathrm{GE}V_{k}=\frac{\sum_{t\in k}(\mathrm{GF}P_{t}^{2}\cdot r_{t,k}^{2})}{\sum_{t}\mathrm{GF}P_{t}^{2}}$$

Where $\mathrm{GF}P_{t}$ denotes the Global Field Power at time point *t* and $r_{t,k}$ represents the spatial correlation between the EEG topography and microstate class *k* (Murray et al., 2008). GEV was selected as the primary metric because it captures both the spatial stability and the temporal contribution of each microstate. GEV represented the proportion of variance in the EEG signal explained by each canonical microstate map and reflects both the temporal contribution and spatial stability of the microstates. Because the present study aimed to evaluate how strongly participants’ EEG dynamics conformed to canonical microstate templates, GEV was used as the primary index of microstate representation. In addition, complementary temporal parameters including mean duration, occurrence, and coverage were analyzed to provide a more comprehensive characterization of microstate dynamics.

### Statistical analysis

Statistical analyses were performed using Jamovi version 2.6.44. Data normality was verified via Shapiro–Wilk tests. Group comparisons for demographic and clinical variables were performed using independent samples t-tests or Mann–Whitney U tests, as appropriate. Microstate GEV differences were evaluated using Mann–Whitney U tests to ensure statistical robustness. Temporal parameters (Duration, Occurrence, and Coverage) were analyzed using MANOVA (Pillai’s Trace), followed by univariate follow-up tests for significant multivariate effects. Associations between microstates and cognitive scores (MMSE, MoCA, RPM, and BFRT) were examined using partial Spearman correlations, adjusted for age, sex, and education. All analyses were corrected for multiple comparisons using the Benjamini-Hochberg FDR procedure (*p* < 0.05).

## Results

### Demographic and neuropsychological comparisons

As shown in Table 1, the MCI and HC groups were comparable in basic demographics. There was no significant difference in age between MCI patients and HC (65.47 ± 8.54 vs. 64.50 ± 7.42 years; *p* = 0.642), nor in sex distribution (15 m/15f for both groups; *p* = 1.000). Although the HC group had a higher mean for years of education (9.43 ± 4.60) compared to the MCI group (7.03 ± 3.61), this difference was not statistically significant (*p* = 0.051).

In contrast, the MCI group performed significantly worse on all neuropsychological assessments. On the MMSE, MCI patients scored significantly lower than HC (25.07 ± 2.60 vs. 28.90 ± 1.24; *p* < 0.001). Similarly, MoCA scores were markedly reduced in the MCI group (17.30 ± 2.61) relative to HC (23.73 ± 2.46; *p* < 0.001). MCI participants also demonstrated significant impairments in domain-specific tasks, scoring lower on the BFRT (7.20 ± 2.83 vs. 9.63 ± 2.30; *p* < 0.001) and the RPM test (15.10 ± 6.48 vs. 26.37 ± 10.77; *p* < 0.001). All cognitive differences were statistically significant (*p* < 0.01) with large effect sizes, confirming the expected global and domain-specific cognitive impairments in the MCI cohort.

### Global explained variance of microstate classes

**Figure 1:**
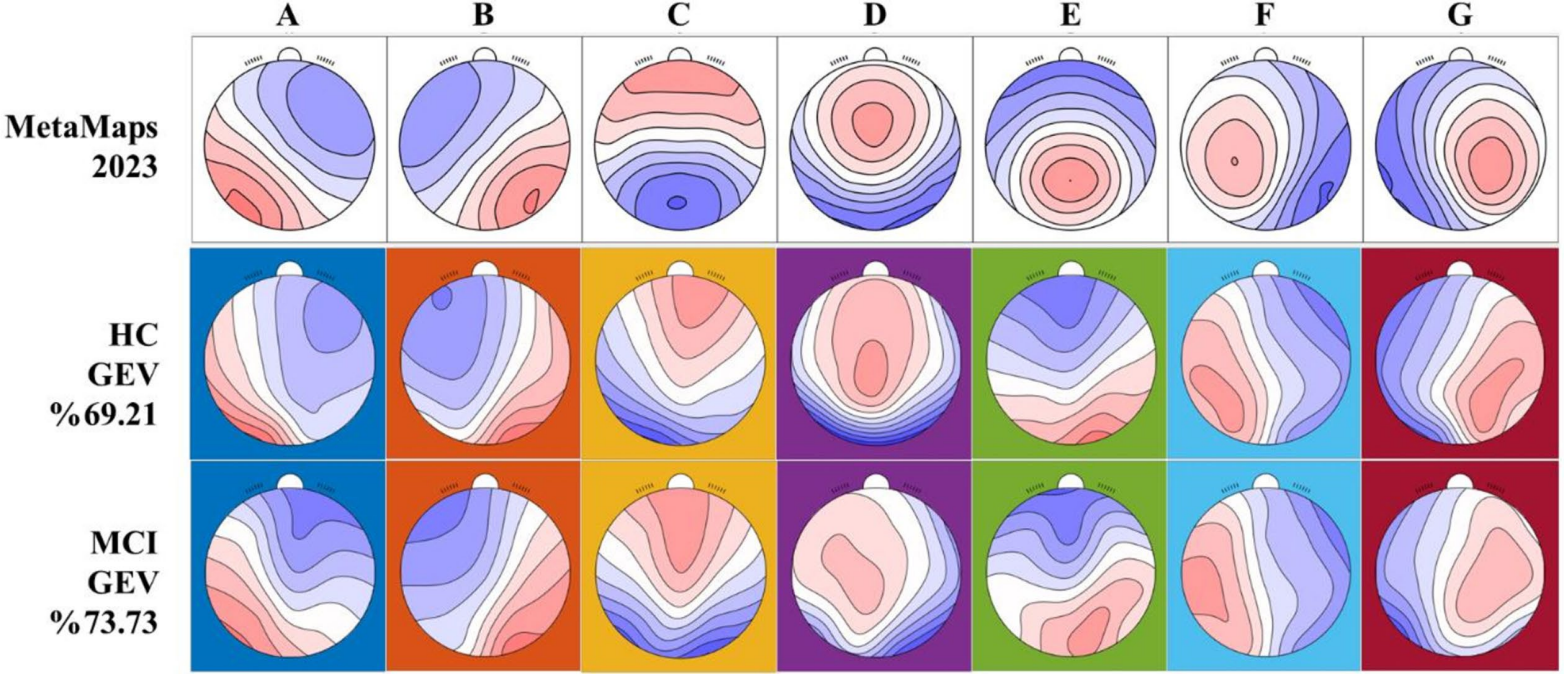
Topographies of EEG microstate classes **(A–G)**. The top row shows the canonical Metamaps2023 templates (Metamaps2023; Koenig et al., 2024) used as spatial references. The middle and bottom rows display the group-averaged microstate maps for healthy controls (HC) and patients with mild cognitive impairment (MCI), respectively. Microstate labels were assigned based on spatial correlation with the MetaMaps2023 templates, supported by visual inspection of topographic similarity. GEV, Global explained variance (%). Corresponding spatial correlation coefficients are provided in Supplementary Table S2.

GEV was calculated for each of the seven microstate classes based on study-specific group templates derived from our dataset. As illustrated in Figure 1, the Metamaps2023 templates (top row) were utilized solely as a spatial reference for labeling the microstate maps empirically identified in the HC (middle row) and MCI (bottom row) groups. The total GEV reached 69.21% in the HC group and 73.73% in the MCI group.

**Figure 2:**
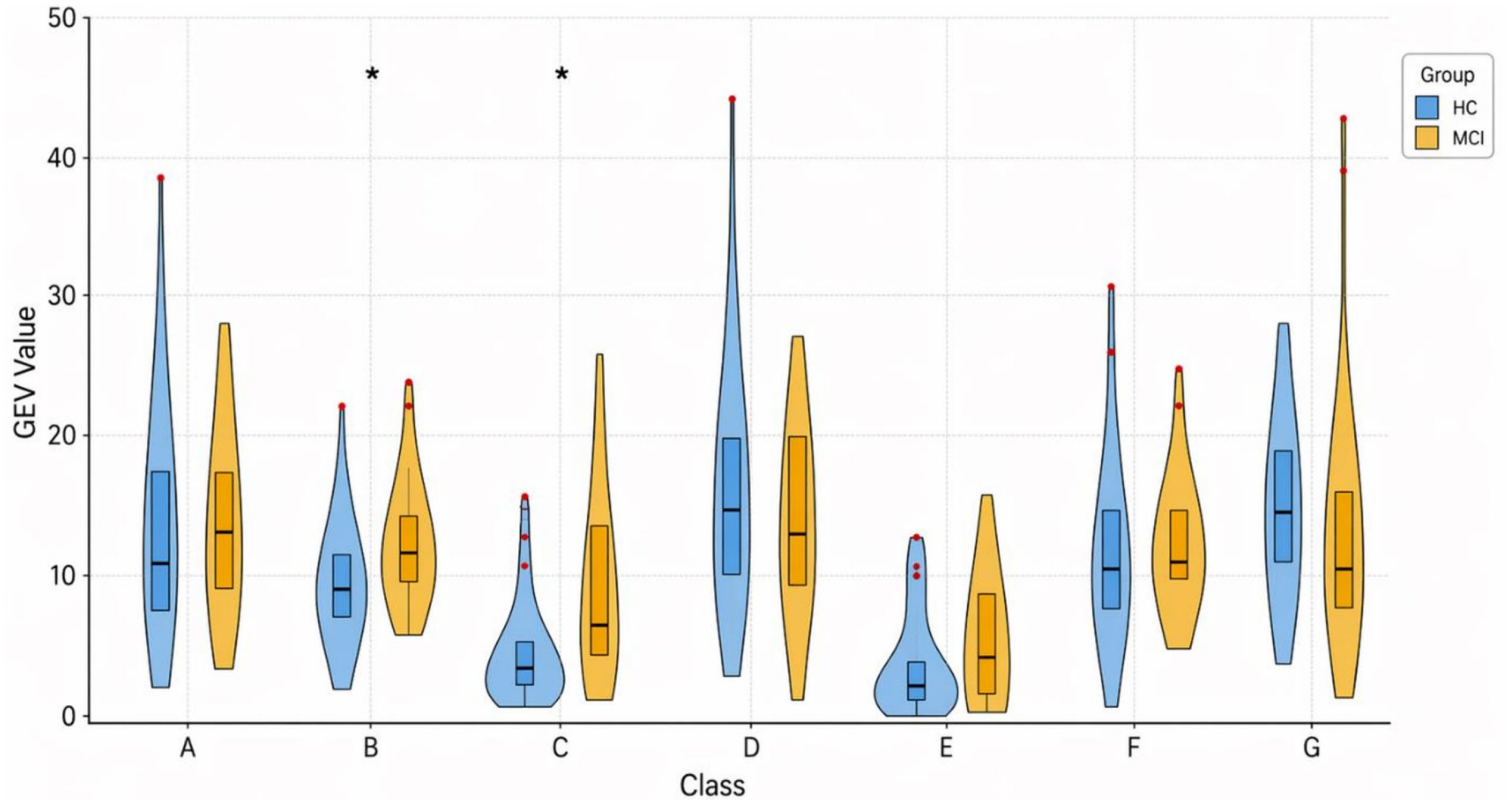
Comparison of GEV values between healthy control (HC) and mild cognitive impairment (MCI) groups across different classes. The violin plots display the smoothed probability density of the GEV distributions for each group. Embedded boxplots show the median (solid horizontal line), interquartile range (box boundaries), and outliers (red dots). Statistical differences between the HC and MCI groups within each class were analyzed using the Mann–Whitney U test. Statistically significant differences are indicated by an asterisk (*p ≤ 0.05).

Analysis of individual microstate contributions revealed significant alterations in classes B and C (Table 2). Specifically, microstate B exhibited a significantly higher GEV in the MCI group compared to healthy controls (HC) (mean GEV ≈ 11.3% vs. 7.9%; *p* = 0.007, Mann–Whitney U test), and microstate C also showed increased GEV in MCI relative to HC (mean GEV ≈ 6.1% vs. 4.1%; *p* = 0.007, Mann–Whitney U test). These findings indicate that microstates B and C contributed more strongly to the overall EEG global variance in individuals with MCI. To facilitate visual interpretation of these differences, the distribution of GEV values for microstates B and C is illustrated using violin plots (Figure 2), while detailed descriptive statistics are shown in Supplementary Table S1.

**Table 2:** Data are presented as mean ± standard deviation.

%	HC (*n* = 30)	MCI (*n* = 30)	*P* value	Effect Size
GEV A	13.39 ± 8.03	11.66 ± 4.97	0.321^a^	−0.1511^b^
GEV B	7.91 ± 4.37	11.32 ± 4.64	**0.007** ^a^	**0.4044** ^b^
GEV C	4.10 ± 3.08	6.09 ± 2.75	**0.007** ^a^	**0.4022** ^b^
GEV D	12.90 ± 7.57	13.47 ± 8.98	0.889^a^	−0.0222^b^
GEV E	1.55 ± 1.12	1.98 ± 1.32	0.116^a^	0.2378^b^
GEV F	6.42 ± 3.80	7.31 ± 2.94	0.134^a^	0.2267^b^
GEV G	8.13 ± 4.47	6.16 ± 4.47	0.053^a^	−0.2911^b^

GEV, Global Explained Variance; HC, Healthy Controls; MCI, Mild Cognitive Impairment.

a *p*-values were calculated using the Mann–Whitney U test to ensure statistical consistency across all microstate classes, as requested during the peer-review process.

b Effect sizes are reported as Rank-Biserial Correlation, which is the appropriate non-parametric effect size for the Mann–Whitney U test. Significant *p*-values (*p* < 0.05) are indicated in bold.

### Microstate temporal parameters

To further characterize microstate dynamics, temporal parameters including mean duration, occurrence, and coverage were analyzed using multivariate analysis of variance (MANOVA). The multivariate analysis revealed significant group differences for microstate classes B and C. Follow-up analyses indicated that these differences were primarily associated with higher occurrence and greater coverage of these microstates in the MCI group, whereas mean duration did not differ significantly between groups (Table 3). Detailed descriptive statistics for duration, occurrence, and coverage across all microstate classes are provided in Supplementary Table S2.

**Table 3:** Temporal dynamics of microstates B and C.

Microstate class	Multivariate test (Pillai’s Trace p)	Univariate: duration (p)	Univariate: occurrence (p)	Univariate: coverage (p)
Class A	0.141	–	–	–
Class B	0.013*	0.100	0.031*	0.006*
Class C	0.021*	0.231	0.008*	0.008*
Class D	0.332	–	–	–
Class E	0.318	–	–	–
Class F	0.191	–	–	–
Class G	0.064	–	–	–

Results from the MANOVA and follow-up univariate tests (Duration, Occurrence, Coverage) are shown. Only classes with significant multivariate effects are detailed. *p < 0.05. Bold values indicate statistical significance (*p* < 0.05).

### Correlation between microstate characteristics and neuropsychological evaluations

After controlling age and education, partial Spearman correlation analyses revealed several significant associations between the individual explained variance of specific microstates and cognitive test scores. Microstate A showed a positive correlation with MMSE scores (*ρ* = 0.351, *p* = 0.007). Microstate B was negatively correlated with MoCA (ρ = −0.379, *p* = 0.003) and Raven’s Matrices Test (*ρ* = −0.348, *p* = 0.007). Microstate C demonstrated the most extensive negative associations, correlating with MoCA (*ρ* = −0.466, *p* < 0.001), Raven’s Matrices Test (*ρ* = −0.389, *p* = 0.003). Microstate G showed positive correlations with MMSE (*ρ* = 0.352, *p* = 0.007).

**Figure 3:**
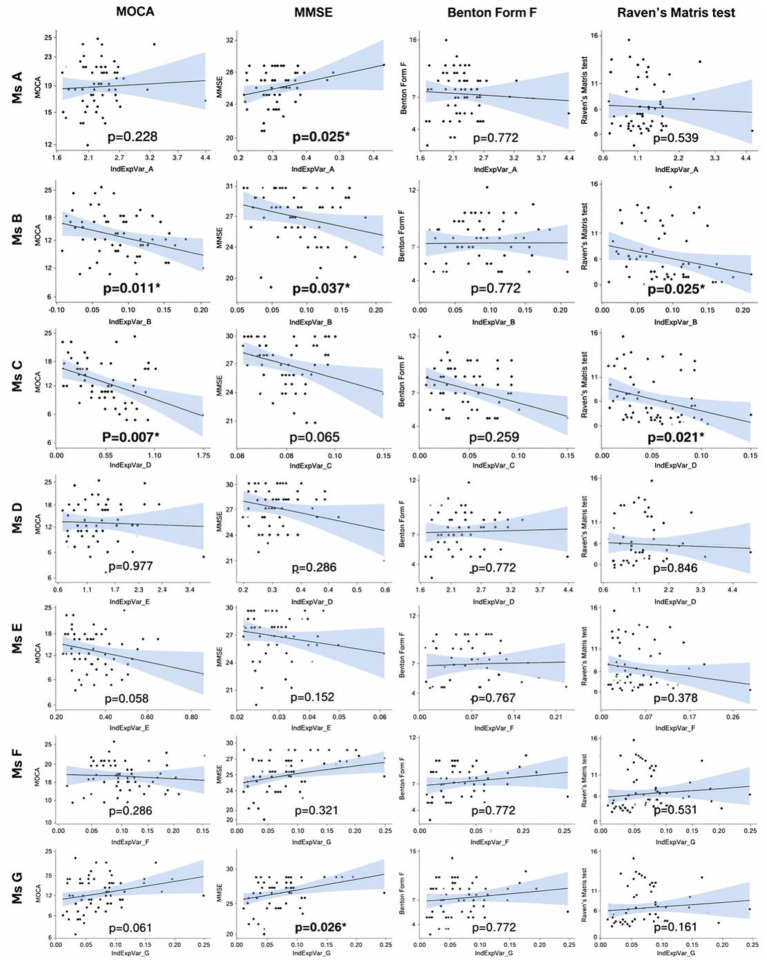
Associations between microstate parameters and cognitive performance. Scatterplots show the relationship between the explained variance of each microstate class (Ms A-G; *x*-axes) and four cognitive outcomes (*y*-axes): MOCA, MMSE, BFRT, and RPM. Lines depict linear fits with 95% confidence bands: each point is a participant. *p*-values are FDR-adjusted (Benjamini-Hochberg) across all contrasts in the figure, asterisks mark *q* < 0.05. Notable patterns include negative associations for Ms. B and Ms. C with MOCA (*p* = 0.011 and *p* = 0.007 respectively) and with Raven’s Matrices (*p* = 0.025 and *p* = 0.021) positive associations for Ms. A and Ms. G with MMSE (*p* = 0.025 and *p* = 0.026), and no significant relationships for BFRT (all *p* = 0.772) Overall, microstate dynamics relate to both global cognitive status (MMSE/MOCA) and higher-order abilities (fluid reasoning on Raven’s), while face recognition (BFRT) shows no microstate dependence in this sample (sample size and recording details as in methods).

These correlations should be interpreted cautiously, as they may partially reflect group differences between MCI patients and HC rather than independent associations between microstate parameters and cognitive performance. No significant correlations were found for microstates D, E, and F with any cognitive measure (Table 4; Figure 3).

**Table 4:** Partial Spearman correlations between microstate parameters and neuropsychological test performance, controlling for age and years of education.

Neuropsychological test	Statistic	IndExpVar_A	IndExpVar_B	IndExpVar_C	IndExpVar_D	IndExpVar_E	IndExpVar_F	IndExpVar_G
MOCA	Spearman’s rho	0.186	−0.379**	−0.466***	−0.004	−0.294*	−0.155	0.277*
	p-value	0.163	**0.003**	**<0.001**	0.977	0.025	0.245	0.035
MMSE	Spearman’s rho	0.351**	−0.314*	−0.274*	−0.155	−0.212	−0.132	0.352**
	p-value	**0.007**	0.016	0.037	0.245	0.109	0.321	**0.007**
Benton Form F	Spearman’s rho	−0.039	−0.054	−0.274	0.080	−0.130	0.135	0.073
	p-value	0.772	0.690	0.037	0.552	0.329	0.312	0.588
Raven’s Matris test	Spearman’s rho	0.099	−0.348**	−0.389**	−0.026	−0.165	−0.111	0.240
	p-value	0.462	**0.007**	**0.003**	0.846	0.216	0.406	0.069

Individual Explained Variance (IndExpVar) refers to the GEV values calculated for each microstate class. *p*-values were corrected for multiple comparisons using the FDR. Bold values indicate statistically significant correlations. *p < 0.05, **p < 0.01, ***p < 0.001 (uncorrected). After FDR correction, significant associations persisted for Classes B and C with MoCA and RPM scores.

## Discussion

Our findings indicate differences in microstate parameters in subjects with MCI, as shown by an increased contribution of microstates B and C compared to healthy controls. However, this finding is not well documented in the literature. Lian et al. (2021), for example, reported an increased contribution of microstate class B in AD but not in MCI; meanwhile, Musaeus et al. (2019) reported an increased contribution of microstate A in MCI, with no differences observed in microstate B states (Lian et al., 2021; Musaeus et al., 2019). Conversely, Lassi et al. (2023) found alterations in the microstate parameters of microstate C in individuals suffering from mild cognitive impairment (MCI) as well as subjective cognitive decline (Lassi et al., 2023).

In the present study, microstates B and C accounted for significantly more variance in the MCI group compared to healthy controls, suggesting that specific large-scale brain networks might be overactive or overrepresented during resting-state in prodromal stages of cognitive decline. Therefore, it is reasonable to suggest that changes in microstate C, which are consistently linked to the default mode network (DMN) and are vital for memory, self-referential thinking, and internal mentation, may reflect either maladaptive reorganization or compensatory hyperactivity within the DMN (Van de Ville et al., 2010). This aligns with the findings of Lassi et al. (2023), who noted that changes in microstate C parameters could potentially signal DMN dysfunction in the early phases of AD.

On the other hand, the negative correlations observed in our study between microstates B and C and MoCA or RPM scores might mean that the overrepresentation of these networks acts as a neural marker of inadequate or maladaptive network integrity (Britz et al., 2010; Michel and Koenig, 2018). However, the positive relationships between microstates A and G and MMSE scores suggest that these states reflect cognitive health and a properly organized neural system. Considering all this, the over-representation of DMN and visual and internally directed networks represented by microstates B and C indicates a disruption in the modulation of large-scale brain networks, supporting the network dysconnectivity model of MCI, which proposes that the brain switches states more frequently to other states but sustains each state for shorter periods (Lassi et al., 2023). Another important contribution to the field is the discovery of the association between the dynamics of the microstate and fluid intelligence in individuals with MCI. As widely known, fluid intelligence reflects the ability for novel problem solving and abstract reasoning, which is not typically considered a memory deficit in amnestic MCI (Ferrer et al., 2009).

An additional point that merits further consideration concerns the differential associations between specific microstate classes and distinct cognitive measures. Increased contribution (GEV) of microstates B and C are linked to lower MoCA and RPM scores, while microstates A and G were associated with better MMSE performance. These findings may reflect the functional specialization of the large-scale networks represented by these microstates. Microstate B has frequently been associated with visual network activity, while microstate C has been linked to the default mode and salience networks involved in internally directed cognition and cognitive control analysis (Khanna et al., 2015). The negative association of these microstates with MoCA and RPM performance may therefore indicate inefficient engagement or dysregulation of networks supporting complex cognitive operations such as abstract reasoning and visuospatial processing.

Our multivariate analysis (Table 3) indicates that the increased GEV of microstates B and C in the MCI group is primarily driven by higher occurrence and coverage, rather than prolonged mean duration. This pattern suggests that these microstates are engaged more frequently over time, without a corresponding increase in their temporal stability. Such alterations in microstate dynamics may reflect changes in large-scale network organization and efficiency. In line with previous literature, increased contributions of microstates associated with salience and visual networks may indicate altered functional processing rather than compensatory mechanisms (Tait et al., 2020). These findings may also be related to reduced cognitive flexibility observed in fluid intelligence measures, supporting the notion that microstate dynamics provide sensitive markers of changes in the temporal organization of brain activity in MCI (Baker et al., 2008).

In contrast, the positive relationship between microstates A and G and MMSE scores may be related to the relative preservation of higher cognitive functions. Microstate A is related to auditory language processing networks, whereas microstate G is related to distributed integrative processes in large-scale brain networks. The different cognitive correlates observed in our study therefore suggest that alterations in microstate dynamics may reflect domain-specific network disruptions in MCI, rather than a uniform global impairment of brain function. This interpretation is consistent with network neuroscience models proposing that early cognitive decline is characterized by selective vulnerability of specific functional systems rather than generalized cortical dysfunction.

In accordance with this approach, Carbone et al. (2024) show that complex higher-order cognitive abilities are independently predictable by resting state microstate dynamics. In this study, this predictive relationship is specific to fluid intelligence (RPM) and executive control (MoCA), indicating that temporal dominance of these microstates acts as a robust marker of the functional integrity of those brain systems critical to abstract thinking processes. Furthermore, the increased dominance of these microstates (Classes B and C) is largely accounted for by increased frequency of these states, as opposed to increased duration (see Table 3). Such a pattern may indicate a potential degradation in network stability and inefficient compensatory mechanisms in increasing switching frequency, a key component in cognitive efficiency (Baker et al., 2008). While microstate dynamics robustly predict fluid intelligence (RPM) and general executive function (MoCA), we did not observe any robust association with BFRT after correction for multiple comparisons. This dissociation suggests that the altered topological dynamics in this MCI cohort are more specifically sensitive to high-level fluid reasoning and dynamic network switching, rather than static, higher order visuoperceptual processing.

Surprisingly, we found significant correlations between microstate parameters and performance on RPM, which prompts us to hypothesize that the dynamics of microstates B and C may be associated with an efficiency cost in cognitive flexibility and problem-solving abilities. Therefore, the idea that spending more time in these states may reflect less efficient transitions between brain networks is supported by the negative correlations observed between the increased explained variance of microstates B and C and RPM and MoCA scores in our current study. Furthermore, this suggests that the disruption of microstates in individuals with MCI is not limited to memory but also impacts fluid cognitive processes such as abstract reasoning and problem-solving, although this has not been consistently confirmed in the literature. For instance, Chenot et al. (2024) stated that there were no significant correlations between resting-state EEG and executive function scores in 140 healthy adults, suggesting that microstate dynamics during normal conditions do not reflect cognitive variability (Chenot et al., 2024). This finding is in contradiction to our MCI group, where the microstate parameters are linked to the measures of fluid reasoning and visuoperceptual performance clearly, with a coupling between microstates and cognition develops after a short decline in network efficiency. These contrasting findings likely stem from differences in methodology and the characteristics of the populations, supporting that the relationship is state-dependent and reflecting early network disorganization unique to MCI.

Our findings are strengthened when the two groups were matched for age and sex, where significant differences in microstate parameters persisted. This pattern suggested that compensatory network mechanisms indicating cognitive maintenance in normal aging may be attenuated in individuals with MCI. To put it in a different way, these findings may indicate that as neural reserve declines, the efficiency of resting-state network coordination may become a stronger determinant of cognitive performance, potentially extending beyond simple memory deficits in MCI associated with specific microstate parameters. For instance, Hatz et al. (2015) found a relationship between certain transitions, especially between class C and A, and MMSE scores in older adults, a novel finding which has been expanded with our results supporting that also higher cognitive abilities, such as fluid reasoning, visual perception, and abstract thinking, might be defective in MCI.

It is noteworthy that our findings may also be regarded as valuable for a biomarker-based perspective. Herein, EEG microstate analysis emerges as a promising temporal biomarker of brain dysfunction in aging and prodromal dementia, as it can capture millisecond-scale fluctuations in large-scale neural network dynamics that are not easily accessible with slower neuroimaging methods such as fMRI or PET. Unlike traditional spectral power measures that collapse signal dynamics into frequency bands (Azami et al., 2023), microstate analysis captures the sub-second stability of global brain states. Hence, it suggests that altered microstate patterns could serve as early indicators of network disruption in groups at risk for dementia. This has been supported by previous literature. For instance, Lassi et al. (2023) identified decreased transition complexity and various microstate irregularities that aligned with the initial functional disruptions seen along the Alzheimer’s continuum. These findings suggest that such irregularities can also be detected in preclinical stages linked to specific cognitive domains, including fluid reasoning and visuoperceptual skills.

Although this research has provided new evidence, it has several limitations to consider. For a relatively small group of participants (*n* = 60), it is possible that generalizability is limited, and larger groups are needed to confirm microstate variations. Additionally, the cross-sectional design is also limited in establishing causality and requires longitudinal studies to determine if microstate disturbances precede the transition from MCI to dementia. Additionally, our cognitive assessment focused on two non-memory measures, leaving other domains such as executive function, attention, and language unexplored. The implications of this are that we should undertake larger and more diverse neuropsychological tests along with different imaging methods like EEG, fMRI, and PET to acquire a clear picture of the brain circuits that get mostly affected during MCI. Besides, there was still a possibility of contamination from factors like medication, vascular diseases, and variable consciousness levels. Nonetheless, our findings indicate that EEG microstates have potential as markers for cognitive impairment across various domains in MCI, and therefore, larger, longitudinal, and multimodal studies are needed to confirm their predictive value and clinical usefulness. Despite these limitations, our data indicates that EEG can effectively capture network correlates across multiple cognitive domains, including fluid intelligence, abstract reasoning, visual recognition, and global measures (such as the MMSE) in MCI.

In that context, individuals with MCI who exhibit significant microstate disruptions may possess relatively diminished cognitive reserves or experience pervasive cortical disorganization, placing them at an elevated risk of progressing to Alzheimer’s dementia. From a network-dynamics perspective, these observations also support the hypothesis that healthy fluid reasoning abilities require the brain’s ability to allow distributed networks to switch in a dynamic manner which again indicates the role of cognitive flexibility in both healthy and diseased states. Our study suggests a shift in perspective for future work by suggesting that the prognostic utility of EEG is not solely dependent on detecting disease, but on tracking the relationship between network dynamics and multidimensional cognitive decline. Integrating multi-domain assessments with microstate features could create a more sensitive, inexpensive, and scalable screening tool that outperforms traditional EEG power measures in early MCI detection (Rossini et al., 2022; Dunstan et al., 2024). Future longitudinal and multimodal studies that will assess their predictive potential are imperative for the practical implementation of these findings in the identification and tracking of dementia risk. These findings highlight the importance of considering microstate specific network contributions when interpreting the neurophysiological correlations of cognitive decline in prodromal dementia.

## Data Availability

The raw data supporting the conclusions of this article will be made available by the authors, without undue reservation.
