# Supplementary material for: Resting-state EEG microstates link neural dynamics to fluid intelligence in mild cognitive impairment
Source: Front Aging Neurosci. 2026 May 20;18:1734828. doi: 10.3389/fnagi.2026.1734828 (PMC13230177; doi:10.3389/fnagi.2026.1734828)
Supplement: Supplementary file 1 [file Data_Sheet_1.docx]

| \| \| **Microstate Class** \| **HC (r)** \| **MCI (r)** \| \| --- \| --- \| --- \| \| A \| 0.82 \| 0.91 \| \| B \| 0.83 \| 0.93 \| \| C \| 0.53 \| 0.59 \| \| D \| 0.88 \| 0.59 \| \| E \| 0.41 \| 0.60 \| \| F \| 0.71 \| 0.71 \| \| G \| 0.86 \| 0.81 \| \| \| --- \| --- \| --- \| --- \| --- \| --- \| --- \| --- \| --- \| --- \| --- \| --- \| --- \| --- \| --- \| --- \| --- \| --- \| --- \| --- \| --- \| --- \| --- \| --- \| --- \| |
| --- | --- | --- | --- | --- | --- | --- | --- | --- | --- | --- | --- | --- | --- | --- | --- | --- | --- | --- | --- | --- | --- | --- | --- | --- | --- |

**Supplementary Table S1. Spatial correlation coefficients (r) between group-averaged microstate maps and MetaMaps2023 templates**

Values represent spatial correlation coefficients (r) between group-averaged microstate topographies (A–G) and MetaMaps2023 reference templates. Higher values indicate stronger spatial correspondence. Microstate labels were assigned based on spatial correlation with the reference templates, supported by visual inspection of topographic similarity.
