# Supplementary material for: Resting-state EEG microstates link neural dynamics to fluid intelligence in mild cognitive impairment
Source: Front Aging Neurosci. 2026 May 20;18:1734828. doi: 10.3389/fnagi.2026.1734828 (PMC13230177; doi:10.3389/fnagi.2026.1734828)
Supplement: Supplementary file 2 [file Data_Sheet_2.docx]

| **Microstate Class** | **Parameter** | **HC (n=30) (Mean ± SD)** | **MCI (n=30) (Mean ± SD)** | **Multivariate F** | **Multivariate p-value** | **Univariate F** | **Univariate p-value** |
| --- | --- | --- | --- | --- | --- | --- | --- |
| **Class A** |  |  |  | 1.90 | 0.141 |  |  |
|  | Duration (ms) | 39.50 ± 12.30 | 38.60 ± 10.03 |  |  | - | - |
|  | Occurrence (hz) | 5.83 ± 1.96 | 5.30 ± 1.48 |  |  | - | - |
|  | Coverage (%) | 22.59 ± 9.10 | 19.60 ± 5.61 |  |  | - | - |
| **Class B** |  |  |  | 3.92 | **0.013** |  |  |
|  | Duration (ms) | 33.40 ± 6.68 | 37.10 ± 10.13 |  |  | 2.80 | 0.100 |
|  | Occurrence (hz) | 4.25 ± 1.34 | 5.07 ± 1.54 |  |  | 4.86 | **0.031** |
|  | Coverage (%) | 14.29 ± 5.15 | 18.03 ± 5.08 |  |  | 8.03 | **0.006** |
| **Class C** |  |  |  | 3.50 | **0.021** |  |  |
|  | Duration (ms) | 29.90 ± 6.10 | 32.40 ± 9.45 |  |  | 1.47 | 0.231 |
|  | Occurrence (hz) | 2.43 ± 0.97 | 3.22 ± 1.24 |  |  | 7.47 | **0.008** |
|  | Coverage (%) | 7.55 ± 3.60 | 10.20 ± 3.87 |  |  | 7.59 | **0.008** |
| **Class D** |  |  |  | 1.16 | 0.332 |  |  |
|  | Duration (ms) | 37.90 ± 8.01 | 39.30 ± 15.45 |  |  | - | - |
|  | Occurrence (hz) | 5.77 ± 1.54 | 5.53 ± 1.65 |  |  | - | - |
|  | Coverage (%) | 21.71 ± 7.68 | 21.09 ± 9.51 |  |  | - | - |
| **Class E** |  |  |  | 1.20 | 0.318 |  |  |
|  | Duration (ms) | 26.00 ± 5.83 | 26.60 ± 5.51 |  |  | - | - |
|  | Occurrence (hz) | 1.96 ± 1.02 | 2.26 ± 1.62 |  |  | - | - |
|  | Coverage (%) | 5.37 ± 3.72 | 5.92 ± 3.49 |  |  | - | - |
| **Class F** |  |  |  | 1.64 | 0.191 |  |  |
|  | Duration (ms) | 30.20 ± 5.09 | 30.60 ± 6.98 |  |  | - | - |
|  | Occurrence (hz) | 4.35 ± 1.57 | 4.66 ± 1.61 |  |  | - | - |
|  | Coverage (%) | 13.20 ± 4.75 | 13.83 ± 3.80 |  |  | - | - |
| **Class G** |  |  |  | 2.56 | 0.064 |  |  |
|  | Duration (ms) | 30.70 ± 7.36 | 29.30 ± 8.77 |  |  | - | - |
|  | Occurrence (hz) | 4.92 ± 1.76 | 3.84 ± 1.39 |  |  | - | - |
|  | Coverage (%) | 15.31 ± 6.11 | 11.34 ± 5.49 |  |  | - | - |

**Supplementary Table 2:** Detailed Comparison of Microstate Parameters Between HC and MCI Groups

Note: HC = Healthy Controls, MCI = Mild Cognitive Impairment, SD = Standard Deviation. Significant p-values (p < 0.05) are indicated in bold. Multivariate statistics reflect Pillai’s Trace from the MANOVA model (df=3, 56) for each respective microstate class. Following the protected ANOVA framework, univariate F and p-values are detailed only for classes exhibiting a significant overall multivariate effect (Classes B and C). Univariate outcomes for non-significant models are intentionally omitted (-).
